# RNA sequencing analysis revealed differentially expressed genes and their functional annotation in porcine *longissimus dorsi* muscle affected by dietary lysine restriction

**DOI:** 10.3389/fvets.2023.1233292

**Published:** 2023-11-08

**Authors:** Md. Shamimul Hasan, Ying Wang, Jean M. Feugang, Huaijun Zhou, Shengfa F. Liao

**Affiliations:** ^1^Department of Animal and Dairy Sciences, Mississippi State University, Starkville, MS, United States; ^2^Department of Animal Science, University of California, Davis, Davis, CA, United States

**Keywords:** lysine, skeletal muscle, RNA sequencing, transcriptomics analysis, swine

## Abstract

The objective of this study was to investigate the effects of dietary lysine restriction on the global gene expression profile of skeletal muscle in growing pigs. Twelve crossbred (Yorkshire × Landrace) barrows (initial BW 22.6 ± 2.04 kg) were randomly assigned to two dietary treatments (LDD: a lysine-deficient diet; LAD: a lysine-adequate diet) according to a completely randomized experiment design (*n* = 6). After feeding for 8 weeks, skeletal muscle was sampled from the *longissimus dorsi* of individual pigs. The muscle total RNA was isolated and cDNA libraries were prepared for RNA sequencing (RNA-Seq) analysis. The RNA-Seq data obtained was then analyzed using the CLC Genomics Workbench to identify differentially expressed genes (DEGs). A total of 80 genes (*padj* ≤ 0.05) were differentially expressed in the *longissimus dorsi* muscle of the pigs fed LDD vs. LAD, of which 46 genes were downregulated and 34 genes were upregulated. Gene Ontology (GO) analysis of the DEGs (*padj* ≤ 0.05) for functional annotation identified those GO terms that are mostly associated with the molecular functions of structural molecules and metabolic enzymes (e.g., oxidoreductase and endopeptidase), biological process of acute-phase response, and amino acid metabolism including synthesis and degradation in the extracellular matrix region. Collectively, the results of this study have provided some novel insight regarding the molecular mechanisms of muscle growth that are associated with dietary lysine supply.

## 1. Introduction

Lysine, an essential nutrient for swine, is typically the first limiting amino acid (AA) in those common grain-based swine diets. Studies have shown that dietary deficiency in lysine can lead to reduced growth performance in pigs due to decreased body protein synthesis and increased fat deposition (1, 2). Therefore, in the swine industry, the pig diets are commonly supplied with the non-protein-bound free lysine (called crystalline lysine) to ensure that pigs receive enough lysine for their optimal growth (3).

At the molecular biological level, lysine has regulatory effects on gene expression, which can alter animal metabolic and signaling pathways associated with protein and lipid metabolism (4). For example, Wang et al. (2) reported that dietary lysine deficiency may activate the ubiquitination pathway to increase muscle protein degradation and up-regulate the expression of genes associated with lipid biosynthesis in finishing pigs. Additionally, Jin et al. (4, 5) also found that dietary lysine deficiency inhibited the satellite cell proliferation and reduced protein synthesis via inhibiting the mTOR signaling pathway in the *longissimus dorsi* muscle of weaning pigs. However, the global gene expression profile or the transcriptional responses to the dietary lysine restriction or deficiency has not been reported in growing pigs.

The transcriptome of a specific tissue under a certain environmental, nutritional, and physiological condition comprises all types of RNAs that include coding and non-coding RNAs (6, 7). In recent years, RNA sequencing (RNA-Seq) – using next-generation sequencing technology – has been employed as a powerful tool to perform a comprehensive analysis and quantification of all RNA species expressed in tissues or cells (7–9). The main objective of this study was to use RNA-Seq to investigate the effects of dietary lysine restriction on the global gene expression profile in the *longissimus dorsi* muscle of young growing pigs. The functions of those differentially expressed genes (DEGs) were annotated as well.

## 2. Materials and methods

### 2.1. Animals and dietary treatments

Twelve crossbred (Yorkshire × Landrace) growing barrows (initial BW 22.6 ± 2.04 kg) were purchased from Prestage Farms of Mississippi (West Point, MS), and housed to an environment-controlled swine barn at the Leveck Animal Research Center of Mississippi State University. Pigs were randomly allocated into 12 individual feeding pens and were allowed to acclimatize to the barn environment for 1 week, while a commercial diet for growing pigs was fed (*ad libitum*). Thereafter, the pigs were assigned to 2 dietary treatments (*n* = 6) according to a completely randomized experimental design with pen or pig as experimental unit.

A corn- and soybean meal-based diet (a lysine-deficient diet; LDD) was formulated (Table 1) to meet or exceed NRC (10) recommended requirements for various nutrients including crude protein and various essential AAs but not lysine. A control diet (a lysine-adequate diet; LAD) was formulated by adding L-lysine monohydrochloride (the commonly used commercial form of crystalline lysine) to the LDD at a rate of 0.40%. No effort was made to maintain the constant ratios of other dietary essential AAs relative to lysine. The samples of diets were collected a few times during the feeding trial, mixed, subsampled, and submitted to the Essig Animal Nutrition Laboratory at Mississippi State University for proximate analysis to confirm the contents of major nutrients and energy. Also, the AA composition of the diets were analyzed at an analytical laboratory of Ajinomoto Heartland, Inc. (Chicago, IL). The analyzed nutrients and AA composition of the two experimental diets are presented in Table 2.

**Table 1:** Composition of the two experimental diets fed to the growing pigs (as-fed basis).

Item	Diet^1^	
	LDD	LAD
*Ingredients*, %		
Corn	74.752	74.352
Soybean meal	20.000	20.000
Canola oil	2.400	2.400
L-Lysine-HCl^2^	0.000	0.400
DL-Methionine^3^	0.080	0.080
L-Threonine^2^	0.100	0.100
L-Tryptophan^4^	0.028	0.028
Limestone	0.810	0.810
Dicalcium phosphate	1.400	1.400
Salt	0.180	0.180
Grow/Finish Premix^5^	0.250	0.250
*Major nutrients, calculated*		
Dry matter, %	82.2	82.2
Net energy, kcal/kg	2,386	2,386
SID^6^ crude protein, %	13.2	13.7
SID lysine, %	0.65	0.96
SID methionine, %	0.30	0.30
SID methionine + SID cysteine, %	0.52	0.52
Total calcium, %	0.68	0.68
STTD^6^ phosphorus, %	0.31	0.31
Crude fiber, %	2.05	2.04
Ash, %	2.23	2.23

1 LDD, a lysine-deficient diet; LAD, a lysine-adequate diet. The calculated total lysine contents (as-fed basis) in LDD and LAD were 0.81 and 1.12%, respectively.

2 L-Lysine-HCl (98.5%) and L-threonine (98.5%) were donated from Archer Daniels Midland Co. (Quincy, IL).

3 DL-Methionine (99.0%, Rhodimet, NP 99) was donated from Adisseo USA, Inc. (Alpharetta, GA).

4 L-Tryptophan (99.0%) was donated from Ajinomoto Heartland, Inc. (Chicago, IL).

5 Grow/Finish Premix 5 (G07390N) was donated from Archer Daniels Midland Alliance Nutrition. The calculated mineral and vitamin contents in both diets were (per kg of diet): S, 0.08 g; Cu, 13.5 mg; Fe, 123.8 mg; I, 0.28 mg; Mn, 27.0 mg; Zn, 123.8 mg, Se, 0.30 mg; vitamin A, 4,953 IU; vitamin D3, 594 IU; vitamin E, 26.4 IU; vitamin K, 2.18 mg; vitamin B_2_, 4.95 mg; niacin, 24.8 mg; vitamin B_5_, 19.8 mg; and vitamin B_12_, 22.3 μg.

6 SID, standardized ileal digestible; STTD, standardized total tract digestible.

**Table 2:** Analyzed nutrient composition (%, or as indicated) of the two experimental diets fed to the growing pigs (as-fed basis).

Item	Diet^1^	
	LDD	LAD
*Proximate and energy analyses* ^2^		
Dry matter	87.8	87.9
Gross energy, kcal/kg	3,946	3,925
Crude protein	15.2	15.2
Ether extract (crude fat)	3.26	3.25
Crude fiber	1.82	1.78
Neutral detergent fiber	13.5	11.3
Acid detergent fiber	2.58	2.28
Ash	4.22	3.97
*Amino acid analyses*^3^, nmol/mL		
Alanine	0.836	0.815
Arginine	1.015	0.987
Aspartic acid	1.579	1.524
Cysteine	0.268	0.268
Glutamic acid	2.789	2.695
Glycine	0.667	0.642
Histidine	0.396	0.383
Isoleucine	0.672	0.643
Leucine	1.369	1.329
Lysine	0.818	1.048
Free lysine	0.158	0.151
Methionine	0.337	0.323
Methionine + Cysteine	0.605	0.591
Phenylalanine	0.788	0.766
Proline	0.937	0.930
Serine	0.781	0.761
Threonine	0.680	0.659
Tyrosine	0.392	0.377
Valine	0.745	0.714
Tryptophan	0.195	0.193

1 LDD, a lysine-deficient diet; LAD, a lysine-adequate diet. The calculated total lysine contents (as-fed basis) in LDD and LAD were 0.81 and 1.12%, respectively.

2 Proximate and energy analyses were conducted at the Essig Animal Nutrition Laboratory, Mississippi State University (Starkville, MS).

3 Amino acid analyses were conducted at the Ajinomoto Heartland, Inc. (Chicago, IL).

### 2.2. Animal trial and sample collection

The animal feeding trial lasted for 8 weeks, and the pigs had *ad libitum* access to the experimental diets and fresh water during the 8-week period. All the pigs, feeders, and waterers were checked 2 to 3 times daily (0600 to 2000 h). At the end of the trial, the pigs were slaughtered in the Meat Science and Muscle Biology Laboratory of Mississippi State University. Skeletal muscle samples (approximately 2 g/pig) were collected from the middle portion of *longissimus dorsi* (between the 10th and 12th ribs) of each pig, and immediately snap frozen in liquid nitrogen. The frozen muscle samples were then stored at −80°C freezer until the RNA-Seq analysis was started. All the animal-related experimental protocols (e.g., caring, handling, and treatment of pigs) were approved by the Mississippi State University Institutional Animal Care and Use Committee (IACUC).

### 2.3. mRNA isolation, and cDNA library construction and sequencing

Total RNA was extracted from the frozen muscle samples using TRIzol Reagent (Invitrogen Corporation, Carlsbad, CA) according to the manufacturer’s instructions. The RNA samples were further purified by DNase I (Ambion, Austin, TX) treatment to avoid any DNA contamination. Thereafter, the RNA integrity (RIN) of the purified RNA samples was assessed using Agilent 2100 Bioanalyzer (Agilent Technologies, Santa Clara, CA), and only the samples with RIN values ≥7 were used for cDNA library preparation. The concentrations and purity of the RNA samples were also determined using a NanoDrop ND-1000 spectrophotometer (NanoDrop Technologies, Wilmington, DE).

Following the quality control analyses, a paired-end (i.e., 250 bp) cDNA libraries were constructed for each sample by using the NEBNext^®^ Ultra^™^ Directional RNA Library Prep Kit (New England Biolabs, Inc., Ipswich, MA) following the manufacturer’s protocols. To generate paired-end reads of 100 bp in FASTQ format, a multiplex sequencing of the cDNA libraries was carried out on a HiSeq 4000 sequence analyzer (llumina, San Diego, CA) at the Genome Center of DNA Technologies Core in the University of California, Davis, CA.

### 2.4. Bioinformatics analyses and data interpretation

#### 2.4.1. Sequence mapping and differential gene expression

The raw RNA-Seq data were analyzed using the CLC Genomic Workbench (Version 22.0; Qiagen, Germantown, MD) program. The quality of the sequence reads was first checked by the quality control pipeline with the default program parameters, and the high-quality reads were mapped against the recent version of swine reference genome, *Sus scrofa* 11.1, housed in a National Center for Biotechnology Information (NCBI) genome database.^1^ Then, the gene expression data were normalized by calculating the fragments per kilobase per million mapped reads (FPKM). The DEG analytical tool of the CLC Genomic Workbench was then used to perform statistical analysis, for which a modified ‘Exact Test,’ developed by Robinson and Smyth (11) and incorporated in the Edge R Bioconductor package by Robinson et al. (12), was implemented. The obtained *p*-values were corrected by Benjamini-Hochberg (B-H) multiple testing procedure.

#### 2.4.2. Gene enrichment and functional analysis of differentially expressed genes

For Gene Ontology (GO) analysis, the web-based functional analysis tool, DAVID (Database for Annotation, Visualization, and Integrated Discovery) bioinformatics program, was used to analyze gene enrichment and functional annotation of the DEG. Briefly, a list of DEG names was uploaded and stored in the centralized list manager panel of DAVID. Since the program is a case insensitive tool for all the accessions (i.e., the IDs), the IDs of DEG were firstly converted to DAVID gene IDs; A total of 78 (80) DAVID IDs were used for further functional analysis while *Sus scrofa* genome annotation was selected as the background species.

Reports for the functional annotation chart were generated for Molecular Functions (MF), Biological Processes (BP), Cellular Components (CC), and Kyoto Encyclopedia of Genes and Genomes (KEGG) pathway enrichment. The Fisher Exact Test was used with the DAVID gene IDs, and only the results that met specific criteria (such as a maximum probability of 0.1 and a minimum count of 2) were shown in the chart. The EASE Score threshold, which is a modified Fisher Exact value of *p*, was used for analyzing those enriched genes and pathways.

## 3. Results

### 3.1. Mapping and identification of differentially expressed genes

As shown in Table 3, the RNA-Seq analysis generated approximately 822 million raw reads from the twelve *longissimus dorsi* tissue samples (i.e., 6 pigs in the LAD group and 6 pigs in the LDD group), and these raw sequence data have been submitted to the NCBI Sequence Read Archive (SRA) and are available under the BioProject accession number PRJNA975688. Following the quality control check, approximately 59 million of low quality and ambiguous sequence reads were removed and those resulted clean sequence reads were further used for analysis. The sequence alignment against the pig genome yielded mapping rates from 87.3 to 95.3% of uniquely aligned sequence reads in pairs. The sequence reads aligned in broken pairs and those unmapped were excluded from the further analysis.

**Table 3:** The mapping results of the RNA sequencing (RNA-Seq) reads.

Pig ID	Diet^1^	Total reads	Trimmed reads	Mapped reads	Unmapped reads	Mapping rates
94	LAD	66,694,464	66,693,967	59,740,088	6,953,879	89.6
88	LAD	63,401,312	63,400,887	55,346,560	8,054,327	87.3
89	LAD	77,594,514	77,593,962	73,857,524	3,736,438	95.2
80	LAD	72,393,866	72,393,391	69,001,002	3,392,389	95.3
77	LAD	75,436,870	75,436,403	68,602,996	6,833,407	90.9
83	LAD	67,693,538	67,693,047	61,436,154	6,256,893	90.8
92	LDD	61,539,720	61,539,313	55,008,750	6,530,563	89.4
90	LDD	70,691,402	70,690,823	61,744,870	8,945,953	87.3
81	LDD	74,263,536	74,263,071	65,930,444	8,332,627	88.8
79	LDD	61,976,484	61,976,015	54,862,678	7,113,337	88.5
76	LDD	58,115,506	58,115,103	51,065,416	7,049,687	87.9
82	LDD	72,988,662	72,988,163	66,553,632	6,434,531	91.2

1 LDD, a lysine-deficient diet; LAD, a lysine-adequate diet. The analyzed total lysine contents in LDD and LAD were 0.818 and 1.048%, respectively.

**Figure 1:**
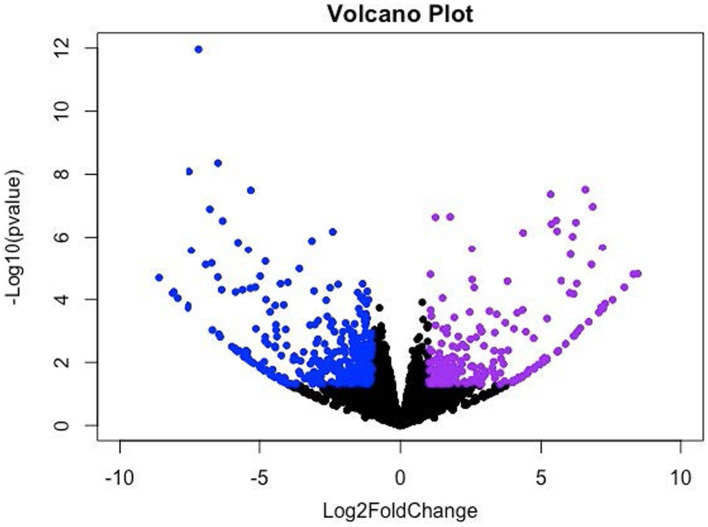
Volcano (scatter) plot for significantly up- and down-regulated genes. *X*-axis and *Y*-axis denote the Log_2_ Fold Change (FC) and −log_10_ of adjusted *p*-values (*padj*), respectively; where −1 ≥ log_2_FC ≥ 1.0 and *padj* < 0.05 were considered significant changes, which are indicated in violet (upregulated) and blue (downregulated) color.

The DEGs were identified using the mode of “against control group” in the CLC Genomic Workbench. This mode tests the differences in gene expression level using “Wald Tests” between the experimental group (i.e., the LDD group) and the control group (i.e., the LAD group). A total of 943 DEGs (*p* ≤ 0.05) were identified which include 101 upregulated genes (Log_2_ Fold Change (FC) ≥ 1) and 337 downregulated genes (Log_2_ FC ≤ −1) in the *longissimus dorsi* muscle of the pigs in the LDD group (Figure 1). Next, the *p*-values were adjusted (*padj*) using the Benjamini-Hochberg (B-H) method. A total of 80 genes (*padj* ≤ 0.05) were found to be differentially expressed due to dietary lysine restriction. The gene names, descriptions, Ensembl identification numbers, and fold changes are listed in Supplementary Table S1.

**Figure 2:**
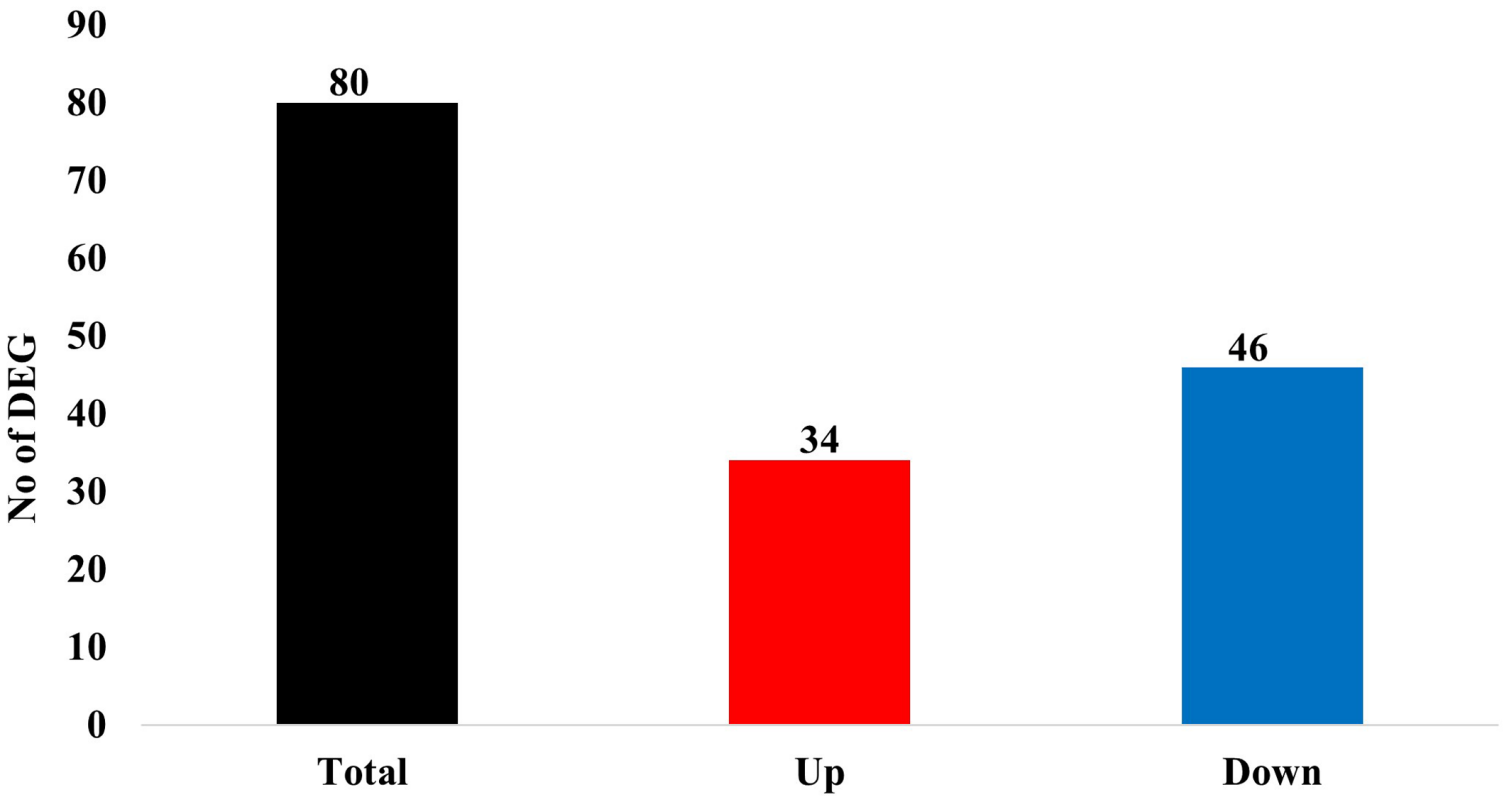
Summary of differentially expressed genes (DEGs) in the skeletal muscle of growing pigs affected by dietary lysine restriction (*padj* ≤ 0.05).

Of these 80 genes, 46 genes were downregulated, and 34 genes were upregulated (Figure 2). The top 25 of the downregulated and top 25 upregulated genes are shown in Tables 4, 5, respectively. The downregulated genes encode various functional proteins, such as transport proteins (TTR, ALB), lipoproteins (APOE, APOC3, APOH), cytochrome p450 family proteins (CYP3A22, CYP2E1), AA metabolic enzymes (PAH, TAT), and fibrinogens (FGA, FGB). On the other hand, the upregulated genes encode those proteins that mainly include structural proteins (Keratin isoforms: KRT 25, 31, 74), proteoglycan (HAPLN1, ACAN), collagen family proteins (COL2A1, COL9A1), and also the enzymes related to AA and energy metabolism (PHGDH, PSAT1).

**Table 4:** The top 25 downregulated genes in the *longissimus dorsi* muscle of the growing pigs fed a lysine-deficient vs. a lysine-adequate diet.

Gene name	Gene symbol	Log₂FC^1^	*padj* ^2^
Transthyretin	TTR	−8.61	0.0096
Histidine rich glycoprotein	HRG	−8.13	0.0183
Cytochrome P450 family 3 subfamily A member 22	CYP3A22	−8.09	0.0177
Glutathione S-transferase alpha 1	GSTA1	−7.57	0.0411
Plasminogen	PLG	−7.55	0.0353
Alpha 2-HS glycoprotein/Fetuin-A	AHSG	−7.51	0.0000
Apolipoprotein H	APOH	−7.42	0.0019
Albumin	ALB	−7.17	0.0000
Cytochrome P450 family 2 subfamily E member 1	CYP2E1	−6.91	0.0045
Apolipoprotein C3	APOC3	−6.76	0.0003
CD163 molecule-like 1	CD163L1	−6.69	0.0043
Fibrinogen gamma chain	FGG	−6.47	0.0000
GC vitamin D binding protein	GC	−6.35	0.0162
Fibrinogen alpha chain	FGA	−6.31	0.0004
Apolipoprotein B	APOB	−5.86	0.0177
Alpha-1-microglobulin/bikunin precursor	AMBP	−5.76	0.0012
Phenylalanine hydroxylase	PAH	−5.60	0.0162
Tyrosine aminotransferase	TAT	−5.33	0.0150
Fibrinogen beta chain	FGB	−5.31	0.0001
Metallothionein-1E	MT1E	−5.15	0.0149
Orosomucoid 1	ORM1	−4.98	0.0090
Hemopexin	HPX	−4.79	0.0039
Carbamoyl l-phosphate synthase 1	CPS1	−4.77	0.0261
C-C motif chemokine ligand 16	CCL16	−4.44	0.0353
Cytochrome P450 family 1 subfamily A member 1	CYP1A1	−4.26	0.0125

1 log_2_ Fold Change (FC) indicates the level of expression: the negative (−) values indicate the downregulation.

2 Adjusted *p*-values (*padj*) were obtained by using the Benjamin-Hochberg multiple testing method provided in the CLC Genomic Workbench (Version 11; Qiagen, Germantown, MD).

**Table 5:** The top 25 upregulated genes in the *longissimus dorsi* muscle of the growing pigs fed a lysine-restricted vs. a lysine-adequate diet.

Gene name^1^	Symbol	Log₂FC^1^	*padj* ^2^
Keratin 74	KRT74	8.48	0.0081
Proline rich 9	PRR9	8.32	0.0081
Keratin 82	KRT82	7.58	0.0261
Keratin 31	KRT31	6.83	0.0045
Keratin 25	KRT25	6.61	0.0001
Keratin 33A	KRT33A	6.31	0.0125
Integrin binding sialoprotein	IBSP	6.28	0.0004
Keratin 27	KRT27	6.16	0.0009
S100 calcium binding protein A3	S100A3	6.09	0.0024
Hyaluronan and proteoglycan link protein 1	HAPLN1	5.57	0.0007
Tenascin N	TNN	5.53	0.0004
Aggrecan	ACAN	5.36	0.0005
Collagen type II alpha 1 chain	COL2A1	5.33	0.0001
Matrix metallopeptidase 13	MMP13	4.36	0.0007
Collagen type IX alpha 1 chain	COL9A1	4.34	0.0446
EGF like repeats and discoidin domains 3	EDIL3	3.80	0.0113
Cartilage oligomeric matrix protein	COMP	3.16	0.0479
Ribonuclease P/MRP subunit p14	RPP14	2.62	0.0149
Phosphoenolpyruvate carboxykinase 2	PCK2	2.55	0.0106
Phosphoglycerate dehydrogenase	PHGDH	2.54	0.0018
Phosphoserine aminotransferase 1	PSAT1	1.77	0.0004
Activating transcription factor 5	ATF5	1.50	0.0243
Exostosin like glycosyltransferase 1	EXTL1	1.25	0.0004
Keratin 80	KRT80	1.08	0.0446
Asparagine synthetase (glutamine-hydrolyzing)	ASNS	1.07	0.0081

1 log_2_ Fold Change (FC) indicates the level of expression: the positive values indicate the upregulation.

2 Adjusted *p*-values (*padj*) were obtained by using the Benjamin-Hochberg multiple testing method provided in the CLC Genomic Workbench (Version 11; Qiagen, Germantown, MD).

### 3.2. Functional annotation of the differentially expressed genes

**Figure 3:**
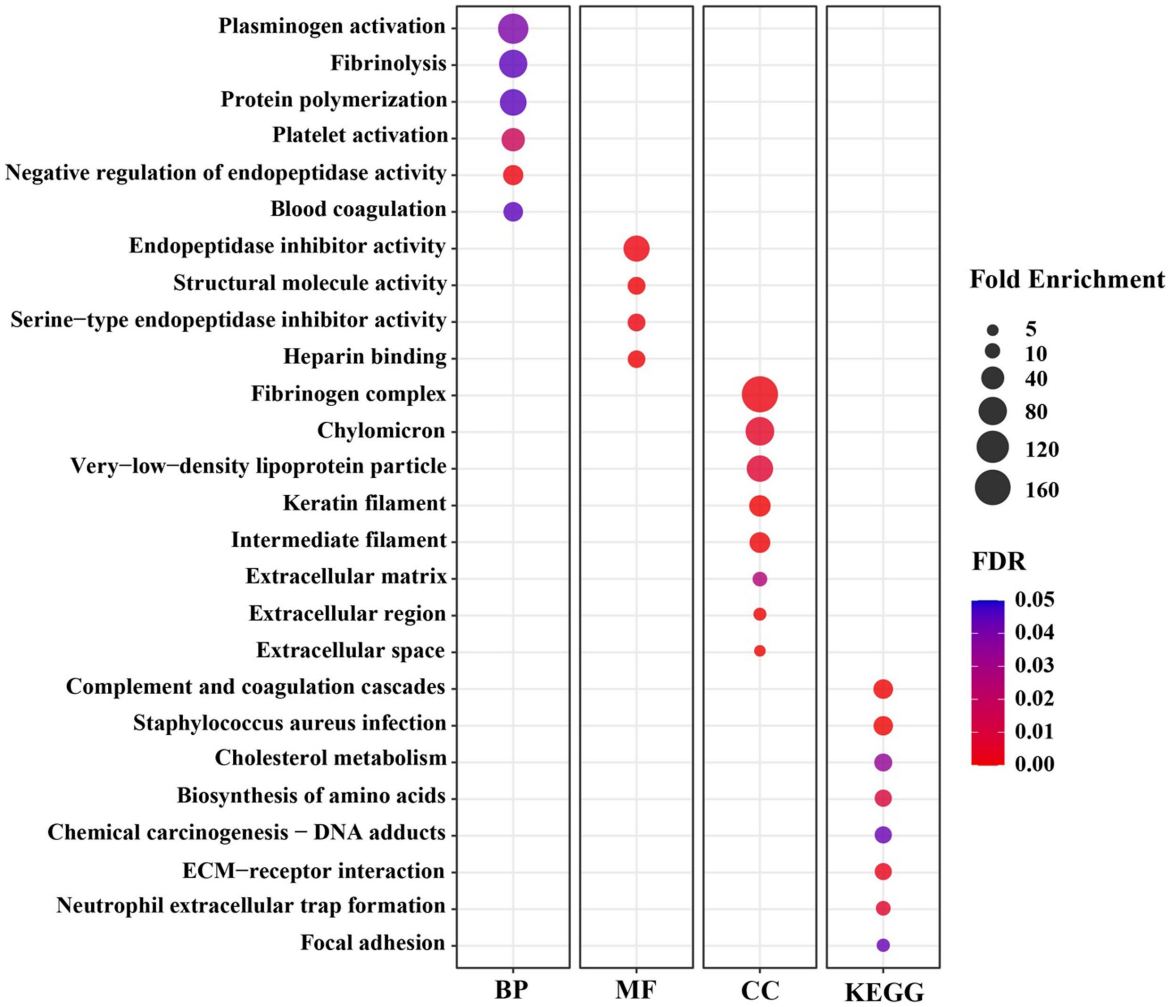
The significant Gene Ontology (GO) terms enriched by differentially expressed genes (DEGs) in the *longissimus dorsi* muscle of growing pigs fed a lysine restricted vs. a lysine adequate diet. BP, Biological Processes; MF, Molecular Functions; CC, Cellular Components; KEGG, Kyoto Encyclopedia of Genes and Genomes.

To understand the biological functions related to those DEGs, a functional annotation analysis was conducted with the DAVID bioinformatics program. The gene accession conversion tool in DAVID was able to convert 78 out of 80 DEGs (97.5%) to DAVID gene IDs. The results of GO analysis of the DEGs demonstrate that the alteration of gene expression levels by dietary lysine restriction may affect the cellular BP, MF, and KEGG pathways. As shown in Supplementary Table S2, the results of gene functional annotation were categorized in MF, BP, CC, and KEGG pathway and described in GO types and terms. The enrichment analysis for MF shows that 60% (47 out of 78) genes are enriched in various MF related GO terms. There were 61 records of MF GO terms found, of which 78% are matched with swine population background. As shown in Figure 3, the major MF-related GO terms include the structural molecule activity, serine-type endopeptidase inhibitor activity, small molecule binding, heme binding, oxidoreductase activity, extracellular matrix (ECM) structural constituent, heparan sulfate proteoglycan binding, and cysteine-type endopeptidases inhibitor activity.

Significantly enriched in different BP-related GO terms were 57% of the DEGs (Figure 3), with the terms including the acute-phase response, cell adhesion, positive regulation of phagocytosis, negative regulation of endopeptidase activity, heterocycle metabolic process, heme transport, L-serine biosynthetic process, growth plate cartilage development, negative regulation of growth, regulation of peptidyl-tyrosine phosphorylation, protein polymerization, high-density lipoprotein particle remodeling, and glutamine metabolic process.

The CC-indicating genes (58% of the DEGs) were detected as highly enriched with the following GO terms (Figure 3): blood microparticle, extracellular space, extracellular region, extracellular exosome, extracellular matrix, keratin filament, intermediate filament, and fibrinogen complex. The KEGG pathway analysis of these 78 DAVID IDs revealed that several cell-signaling and metabolic pathways could be affected by dietary lysine restriction (Figure 3), of which the top 5 are responsible for complement and coagulation cascades, chemical carcinogenesis, biosynthesis of amino acids, metabolism of xenobiotics by cytochrome P450, and steroid hormone biosynthesis pathways.

## 4. Discussion

Lysine is typically the first limiting essential AA in grain-based swine diets. It has been known that dietary deficiency in lysine will negatively affect not only pigs’ muscle – the largest part of the body mass – protein deposition, but also the overall heath and growth performance (1, 2). The growth performance data generated from this study have been reported previously, which showed that the average daily weight gain and the gain to feed ratio were both significantly decreased in the pigs fed LDD than fed LAD, although there was no difference in the average daily feed intake between the two groups of the pigs (3). The reduced weight gain observed in this study must be the consequence of pigs’ poor utilization of dietary nutrients and, especially the proteinogenic AA, for body protein biosynthesis and muscle growth, since the skeletal muscle is the largest organ, as well as the largest protein pool, in the body (1, 3). To further explore the molecular mechanism responsible for the reduced growth (mainly the muscle growth) in response to the dietary lysine restriction, we conducted this global gene expression profiling to identify the DEGs that may have diverse molecular functions associated with the growth and development of the skeletal muscle.

Through this study, we found that dietary lysine restriction downregulated some transporter-protein coding genes (Table 4), such as transthyretin (TTR) and albumin (ALB). Although the TTR and ALB genes are highly expressed in the liver according to the human gene annotation, Soprano et al. (13) and Wagatsuma et al. (14) reported that these genes were also expressed in animal skeletal muscle. The TTR gene product is a 55-kDa homotetraeric transporter protein for T3 and T4 (both are thyroid hormones) in the blood (15). According to Monk et al. (16), TTR deficient mice show a slower transition from suckling to weaning, slower growth, less muscle mass, and impaired bone growth. In addition, some other studies demonstrated that TTR plays a role in promoting the differentiation of muscle cells and the repair of muscle tissue after injury (15, 17, 18). The ALB gene product is another acute phase reactant and a non-specific protein transporter for numerous hormones existing in the blood. It has the highest binding affinity for T3 and T4 followed by TTR, and it determines the transportation time and distribution of thyroid hormones to target tissues. Hypoalbuminemia can be caused by poor nutrition and ongoing inflammation in animals (19). A cross-sectional study reported a significant association between low serum albumin and low muscle strength (20). These results suggest that the reduced skeletal muscle protein turnover and altered local muscle metabolism of thyroid hormones may lead to an intracellular diminished T3 availability (21). Therefore, the downregulation of TTR and ALB genes in this study implies that a reduction in the expression of TTR and ALB genes may be a factor in the negative impact of a lysine restricted diet on muscle growth. This could be due to an impact on those proteins responsible for transporting hormones such as thyroid hormones and other signaling molecules.

The expression of several genes coding for the cytochrome P450 family members, such as cytochrome P450 family 1 subfamily A members 1 and 22 (CYP1A1 and CYP1A22), and cytochrome P450 family 2 subfamily E member 1 (CYP2E1), was decreased in pigs fed the lysine-restriction diet (Table 4). It is known that CYP1A1 and CYP2E1 enzymes metabolize polyunsaturated fatty acids to synthesis biologically active intracellular cell-signaling molecules or lipid mediators, such as ω-3 fatty acid derived resolvins and maresins (22). Some of the lipid mediators are well known for their function in maintaining skeletal muscle mass. During nutritional stress (e.g., deficiency), these anti-inflammatory molecules play important roles in reducing skeletal muscle wasting (23). Consequently, the decreased expression of the genes that are associated with polyunsaturated fatty acid metabolism and synthesis of anti-inflammatory molecules, may negatively contribute to skeletal muscle maintenance and homeostasis.

Of the downregulated genes, phenylalanine hydroxylase (PAH) and tyrosine amino transferase (TAT) are associated with AA metabolism. The PAH enzyme catalyzes the conversion of L-phenylalanine to L-tyrosine, which indicates that dietary lysine restriction may suppress the PAH enzyme activity in pigs. It has long been found that a depressed PAH activity is associated with a decreased ability to dispose the phenylalanine load, leading to an impaired tyrosine formation (24). Tyrosine is used not only in protein synthesis but also in the synthesis of some neurotransmitters (e.g., dopamine, epinephrin, and nor-epinephrin) and hormones (e.g., thyroid hormone). In addition, tyrosine acts as a substrate in reaction with oxoglutarate catalyzed by TAT enzyme to produce 4-hydroxyphenylpyruvate and glutamate. Glutamate can act as a precursor for several neurotransmitters (e.g., GABA) (25). Thus, a decrease in the expression of PAH and TAT in response to dietary lysine restriction may indirectly affect the production of some neurotransmitters and hormones associated with animal growth.

The term “keratin” is known as intermediate filament-forming proteins in cell cytoskeleton, which belong to fibrillar proteins. Keratin fundamentally influences the architecture (e.g., cell polarity and cell shape) and mitotic activity of epithelial cells to sustain their mechanical stress, maintain their structural integrity, and establish cell polarity (26, 27). This study revealed that dietary lysine restriction upregulated the expression of several isoforms of keratin genes including keratin 74 (KRT74), keratin 82 (KRT82), keratin 31 (KRT31), keratin 25 (KRT25), and keratin 33A (KRT33A) (Table 5). As is known, most keratin isoforms are annotated as hair and skin specific in humans, a contamination of our muscle samples by hair or skin tissue is most unlikely due to our rigorous sample preparation protocol. Furthermore, some previous studies had reported the presence of several type I and type II intermediate filament proteins (known as keratins) in developing and striated muscle (28, 29). For example, keratin 19 (K19) and keratin 8 (K8) are prominent keratins expressed in mature striated muscle, where they play a pivotal role in upholding the structural integrity of muscle fibers (30).

There is no previous research, however, that demonstrated a regulatory effect of lysine on keratin gene transcription and translation *in vivo*. It is of interesting in finding that dietary lysine may play a role in keratin structural activity, which warrants further investigation.

The ECM of skeletal muscle – a complex meshwork consisting of collagens, glycoproteins, proteoglycans, and elastin – maintains the tissue structure and integrity (31). The mRNA abundance of several genes that encode collagens, such as collagen type II alpha 1 chain (COL2A1) and collagen type IX alpha 1 chain (COL9A1), proteoglycans, such as hyaluronan and proteoglycan link protein 1 (HAPLN1) and aggrecan (ACAN), and glycoproteins, such as tenascin N (TNN), was increased in this study. The collagen is the major protein in ECM. It has lower fractional synthetic rate than the myofibrillar proteins, and its concentration in the skeletal muscle is only 15 to 20% of the myofibrillar proteins. The suboptimal nutritional condition, such as AA deficiency, may increase in the collagen fractional synthesis rate while decreasing the syntheses of myofibrillar and sarcoplasmic proteins (32). Therefore, the dietary lysine restriction may be a suboptimal nutritional condition that upregulates the collagen synthesis in pigs.

Cartilage proteoglycan link proteins, on the other hand, are the major non-collagenous components of the ECM cartilage (33). In this study, the expression of aggrecan (ACAN) and hyaluronan and proteoglycan link protein 1 (HAPLN1) genes was increased in response to dietary lysine restriction. ACAN is best known as a major, essential, and defining cartilage component quantitatively. According to Mundlos et al. (34), the gene expression patterns of aggrecan and link protein are identical in different tissues, which agrees with our finding. The regulatory mechanism that promotes such drastic alteration in mRNA expression in response to lysine restriction remain uncertain and will be subject to further studies.

The *de novo* biosynthesis of serine and glycine can generate carbon units that satisfy many metabolic demands including nucleotide precursors, redox maintenance, and substrates for methylation reaction (35, 36). Of the several enzymatic reaction steps in serine biosynthesis pathway, phosphoglycerate dehydrogenase (PHGDH) catalyzes the conversion of 3-phosphoglycerate into 3-phosphohydroxypyruvate. Phosphoserine aminotransferase 1 (PSAT1) is a pivotal enzyme that regulates the production of two metabolites, serine, and α-ketoglutarate (α-KG), which are involved in one carbon metabolism and the TCA cycle, respectively (1, 37). This study shows that both PHGDH and PSAT1 genes were upregulated in the *longissimus dorsi* muscle of the pigs fed the lysine restricted diet. This is in agreement with one of our previous studies, which revealed that dietary lysine deficiency decreased the expression of PHGDH and PSAT1 in the *longissimus dorsi* muscle of finishing pigs (2). Overexpression of these genes indicate that dietary lysine level could affect the balance of one carbon metabolism and its associated metabolic pathways.

The widely expressed activating transcription factor (ATF5) regulates cell cycle, differentiation, homeostasis, and survival (38, 39). The results of this study indicate that the expression of ATF5 was increased in pigs fed the LDD diet compared to the LAD diet. Similar findings in previous studies reported that ATF5 expression was increased in response to cellular stress conditions such as AA limitation, heat stress, or oxidative stress (39, 40). Increased activation of ATF5 may upregulate the protein kinases phosphorylation of EIF2α and lead to a global reduction in protein translation.

Six BP terms were significantly enriched under the lysine restriction status (Figure 3), of which protein polymerization (66-fold), fibrinolysis (79-fold), and plasminogen activation (99-fold) were highly enriched. Based on these terms, it can be seen that some DEGs are mostly associated with the homeostasis, wound healing, inflammation, angiogenesis, and several other biological functions (41, 42). For example, fibrinogen, a proteolytic glycoprotein, composed of three closely linked polypeptides coded from FGA, FGB, and FGG genes. Dietary lysine restriction downregulated these genes that may affect the rate of fibrinogen synthesis and result in abnormalities in blood circulation (42), which in turn may indirectly affect the transportation of oxygenated blood or nutrients to the muscle.

Secondly, there were 4 MF terms that were significantly enriched (Figure 3), of which, the highly enriched one was endopeptidase inhibitor activity (62-fold). Endopeptidases, including trypsin, chymotrypsin, elastase, and pepsin, are proteolytic enzymes that break the peptide bonds of non-terminal AAs (43, 44), and are associated with a wide range of biological roles, such as developmental processes, digestion, fertilization, blood coagulation, apoptosis, fibrinolysis, and immune defense (45). The negative effect of dietary lysine restriction on pig growth performance may be also associated with endopeptidase inhibition.

Thirdly, there were 8 CC terms that were significantly enriched (Figure 3), of which the fibrinogen complex was enriched by 166-fold. The CC terms in GO analysis demonstrate the subcellular location or the structure of macromolecular complex of gene products (46). As shown in Figure 3, a high percentage of gene products among the DEGs are part of fibrinogen complex, intermediate filament, and ECM. Fibrinogen, an important component of ECM, is a large complex protein composed of three polypeptide chains, such as A, B, and G that are encoded by FGA, FGB, and FGG genes, respectively (47, 48). The DEG result of this study indicates that the dietary lysine restriction decreased the fibrinogen gene expression (Table 4) that may lead to a reduction in the amount of fibrinogen available to be incorporated into ECM, leading to a weaker ECM and a reduction in the angiogenesis process, which could affect the integrity of ECM and muscle growth (49, 50).

Finally, the results of the GO terms of KEGG pathway enrichment indicate that the negative impact of feeding lysine restricted diet to pigs on the growth may be mediated via the signaling and metabolic pathways that include AA biosynthesis, cholesterol metabolism, receptor interaction, and complement and coagulation cascades (Figure 3). For example, Carbamoyl l-phosphate synthase 1 (CPS1) gene encode an enzyme involved in nitrogen metabolism that removes nitrogen waste products from the body (51). The decrease in CPS1 expression was observed in this study suggesting that it may cause disruption in the balance of muscle protein synthesis and degradation, leading to muscle wasting. It is important to note that AA metabolism is a very complex metabolic process whose regulatory mechanism warrants further investigation.

At the end, authors would like to point out that the mRNA abundance does not always correlate with protein abundance, even though many scientists use mRNA abundance to indicate protein abundance because in many cases more mRNA would translate out more protein. Also, it should be kept in mind that the global gene expression data in general are not easy to be interpreted simply because of the complexities not only in the data themselves but also in the aspect of animal biological processes.

## 5. Conclusion

A transcriptome analysis using RNA-Seq technology revealed that dietary lysine restriction altered the gene expression profile in *longissimus dorsi* muscle of growing pigs. We found that 80 genes were differentially expressed, of which 46 genes were down-regulated and 34 genes were up-regulated. Some of the down-regulated genes (e.g., TTR, ALB) are associated with transportation of signaling molecules (e.g., hormones and neurotransmitters) and AA metabolism (e.g., PAH, TAT). On the other hand, the down-regulated genes, such as keratin, ACAN, and COL isoforms are associated with the cell structural molecule activities and AA metabolism (e.g., PHGDH and PSAT1). The GO analysis results suggest that the dietary lysine restriction may influences some biological processes, such as blood coagulation and inflammation, some molecular functions, such as endopeptidase inhibitory activity; and some KEGG pathways, such as the complement and coagulation pathway. Collectively, the results generated from this study have provided some critical novel insight regarding the molecular mechanisms of muscle growth that are associated with dietary lysine supply.

## Data availability statement

The datasets presented in this study can be found in online repositories. The names of the repository/repositories and accession number(s) can be found in the article/Supplementary material.

## Ethics statement

The animal study was approved by Mississippi State University Institutional Animal Care and Use Committee (IACUC). The study was conducted in accordance with the local legislation and institutional requirements.

## Author contributions

SL conceptualized the study, acquired funding for the investigation, and coordinated the whole project. JF and HZ participated in the experiment design and manuscript preparation. MH and SL conducted the animal trial and analyzed data and prepared the manuscript. MH and YW conducted the laboratory sample analyses. All authors approved the final version of the manuscript.

## Footnotes

^1^ https://www.ncbi.nlm.nih.gov
